# Supplementary figures and images for: Long-term neurological manifestations of COVID-19: prevalence and predictive factors
Source: Neurol Sci. 2021 Sep 15;42(12):4903–7. doi: 10.1007/s10072-021-05586-4 (PMC8439956; doi:10.1007/s10072-021-05586-4)

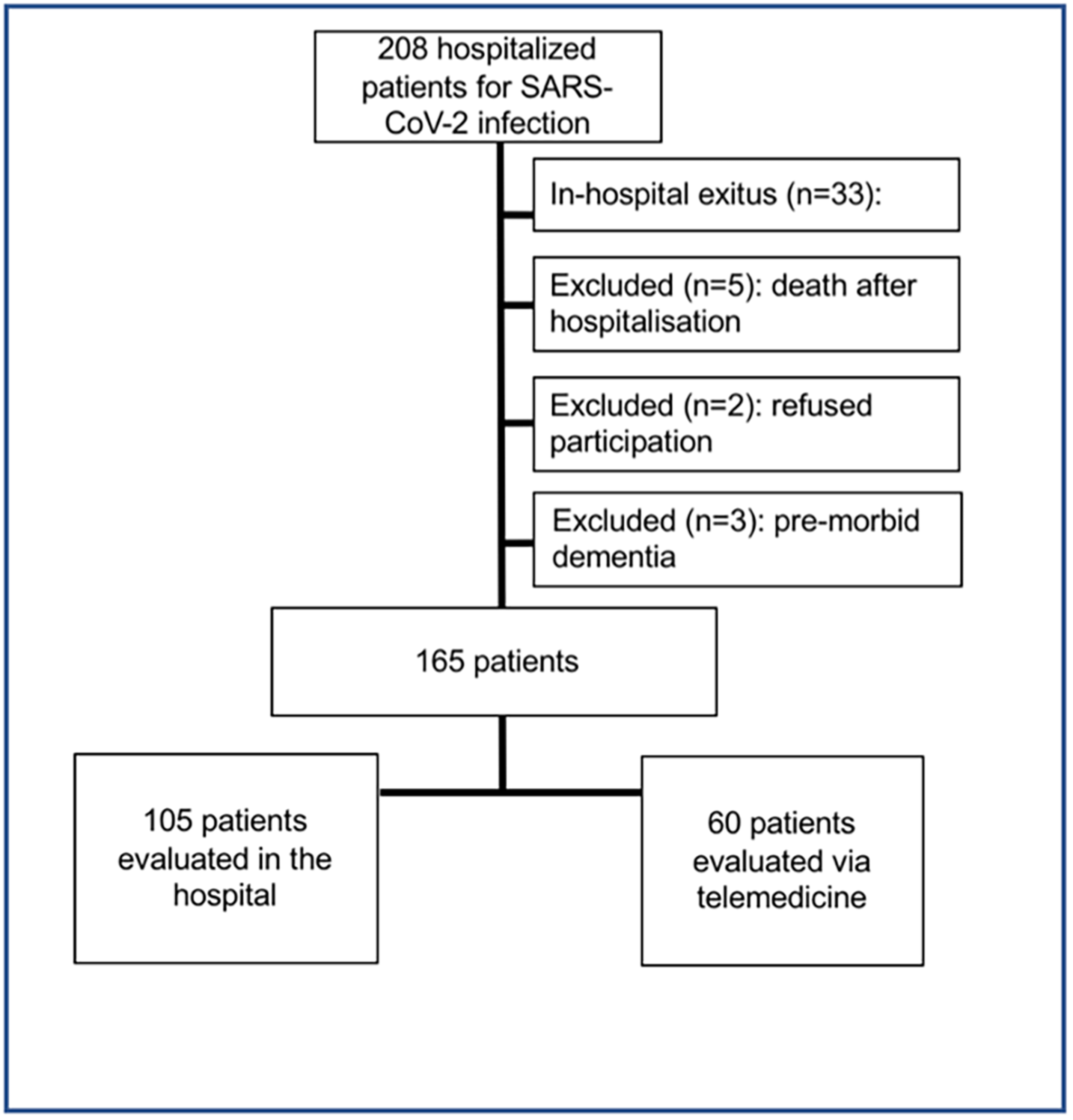

Supplement: Supplementary file 1 — (PNG 675 kb) [file 10072_2021_5586_Fig2_ESM.png]

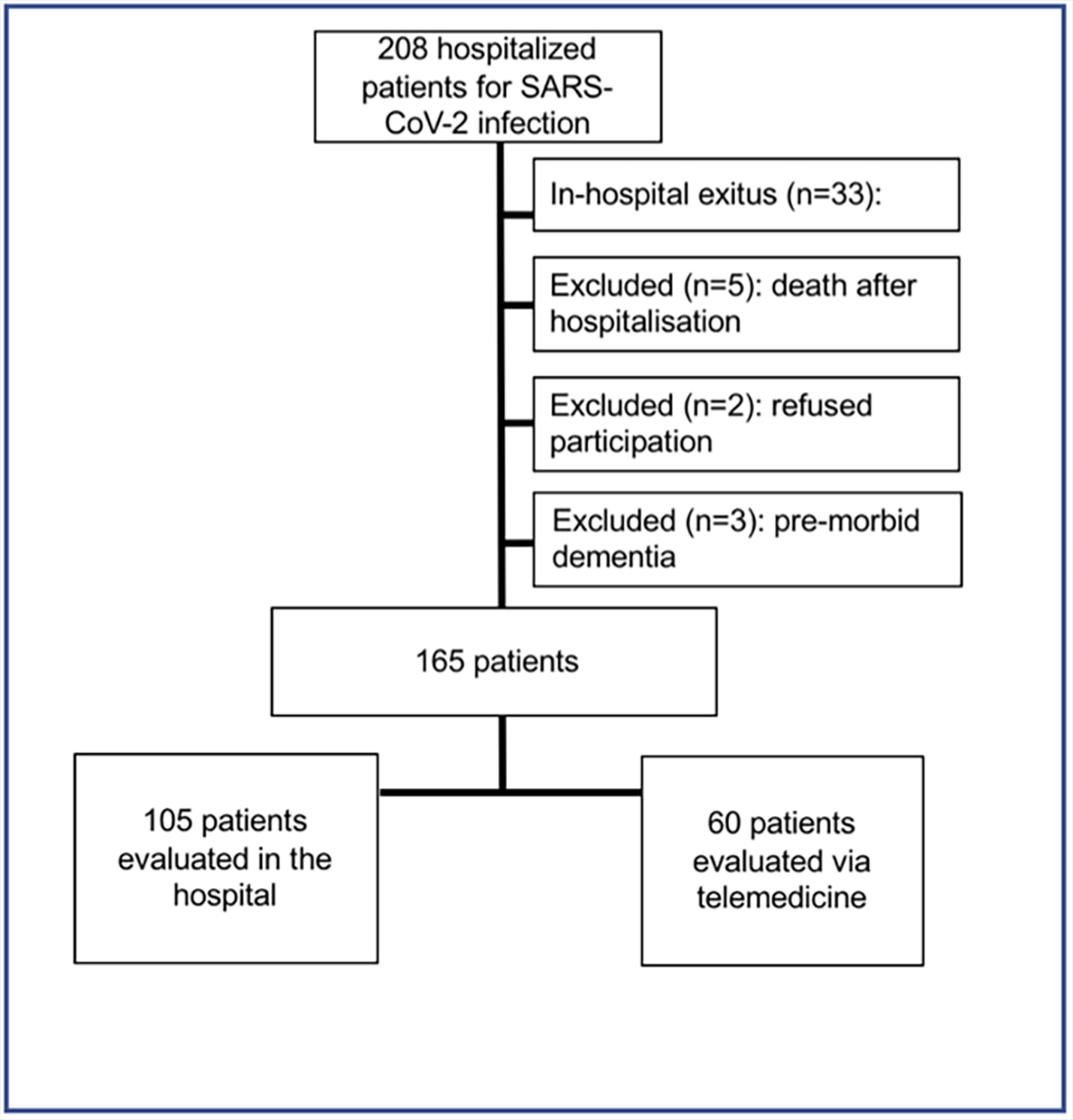

Supplement: Supplementary file 2 — High resolution image (TIF 9135 kb) [file 10072_2021_5586_MOESM1_ESM.tif]
