# Supplementary material for: Long-term neurological manifestations of COVID-19: prevalence and predictive factors
Source: Neurol Sci. 2021 Sep 15;42(12):4903–7. doi: 10.1007/s10072-021-05586-4 (PMC8439956; doi:10.1007/s10072-021-05586-4)
Supplement: Supplementary file 3 — (DOCX 20 kb) [file 10072_2021_5586_MOESM2_ESM.docx]

**Supplementary Table 1 NEURO-COVID19 BRESCIA CHECKLIST**

We kindly ask you to answer all the following questions. ì. The questions refer to the current medical condition ( last 2 weeks) state compared to time before COVID-19 infection.

|  | **Symptoms and Complaints after COVID19** | **NO** | **YES** |
| --- | --- | --- | --- |
| 1 | Memory impairment |  |  |
| 2 | Planning/attention complaints |  |  |
| 3 | Confusion |  |  |
| 4 | Depression/Anxiety |  |  |
| 5 | Hypotension |  |  |
| 6 | Postural instability or falls |  |  |
| 7 | Gait disturbances |  |  |
| 8 | Abnormal movements |  |  |
| 9 | Fatigue |  |  |
| 10 | Numbness or tingling |  |  |
| 11 | Blurring/loss of vision |  |  |
| 12 | Hyposmia/hypogeusia |  |  |
| 13 | Sleep disorders |  |  |
| 14 | Urinary dysfunction |  |  |
| 15 | Swallowing difficulties |  |  |
| 16 | Dysarthria/speech disturbances |  |  |
| 17 | Headache |  |  |
| 18 | Myalgia |  |  |
