# Supplementary material for: Long-term neurological manifestations of COVID-19: prevalence and predictive factors
Source: Neurol Sci. 2021 Sep 15;42(12):4903–7. doi: 10.1007/s10072-021-05586-4 (PMC8439956; doi:10.1007/s10072-021-05586-4)
Supplement: Supplementary file 4 — (DOCX 14 kb) [file 10072_2021_5586_MOESM3_ESM.docx]

**Supplementary Table 2:** Different prevalence of Neurological symptoms reported by patients using the checklist according to different COVID- 19 severity. Abnormal movements included tremor, dystonia, dyskinesia, chorea and all hyperkinetic disorders. Abbreviations: IADL, instrumental activities of daily living

|  | **Total**  **(n=165)** | **Mild**  **(n=57)** | **Moderate**  **(n=77)** | ***Severe***  **(n=31)** | ***p* value** |
| --- | --- | --- | --- | --- | --- |
| Fatigue | 56 (33.9%) | 17 (29.8%) | 24 (31.2%) | 15 (48.4%) | 0.18 |
| Memory/Concentration complaints | 52 (31.5%) | 19 (33.3%) | 16 (20.8%) | 15 (48.4%) | 0.015 |
| Sleep disorders | 52 (31.5%) | 25 (43.8%) | 15 (19.5%) | 12 (38.7%) | 0.006 |
| Myalgia | 50 (30.3%) | 18 (31.6%) | 19 (24.7%) | 12 (38.7%) | 0.319 |
| Depressive symptoms/ anxiety | 44 (26.7%) | 19 (33.3%) | 15 (19.5%) | 10 (32.3%) | 0.134 |
| Dyspnea | 35 (21.2%) | 9 (15.7%) | 17 (22.1%) | 9 (29.0%) | 0.28 |
| Loss of dependency in IADL | 34 (20.7%) | 10 (17.5%) | 13 (16.8%) | 11 (32.4%) | 0.08 |
| Blurring/loss of vision | 32 (19.5%) | 11 (19.3%) | 9 (11.7%) | 12 (38.7%) | 0.006 |
| Numbness/tingling | 31 (18.8%) | 12 (21.4%) | 12 (15.6%) | 7 (22.6%) | 0.58 |
| Hyposmia/hypogeusia | 27 (16.4%) | 7 (12.3%) | 12 (15.6%) | 8 (25.8%) | 0.26 |
| Urinary dysfunction | 23 (13.9%) | 9 (15.8%) | 9 (11.7%) | 5 (16.1%) | 0.72 |
| Confusion | 22 (13.3%) | 7 (12.3%) | 7 (9.1%) | 8 (25.8%) | 0.07 |
| Hypotension | 20 (12.2%) | 10 (17.5%) | 4 (5.2%) | 6 (19.4%) | 0.04 |
| Gait disturbances | 18 (10.9%) | 8 (14.0%) | 6 (7.8%) | 4 (12.9%) | 0.46 |
| Abnormal movements | 17 (10.3%) | 7 (12.3%) | 5 (6.5%) | 5 (16.1%) | 0.67 |
| Headache | 16 (9.7%) | 7 (12.5%) | 5 (6.5%) | 4 (12.9%) | 0.41 |
| Postural instability or falls | 14 (8.5%) | 4 (7.0%) | 5 (6.5%) | 5 (16.1%) | 0.24 |
| Swallowing difficulties | 10 (6.1%) | 4 (7.0%) | 2 (2.6%) | 4 (12.9%) | 0.11 |

p values were calculated by chi-square test.
